# A structured, home-based exercise programme in kidney transplant recipients (ECSERT): A randomised controlled feasibility study

**DOI:** 10.1371/journal.pone.0316031

**Published:** 2025-02-24

**Authors:** Roseanne E. Billany, Jamie H. Macdonald, Stephanie Burns, Rafhi Chowdhury, Ella C. Ford, Zahra Mubaarak, Gurneet K. Sohansoha, Noemi Vadaszy, Hannah M. L. Young, Nicolette C. Bishop, Alice C. Smith, Matthew P. M. Graham-Brown

**Affiliations:** 1 Department of Cardiovascular Sciences, University of Leicester, Leicester, United Kingdom; 2 Institute for Applied Human Physiology, Bangor University, Bangor, United Kingdom; 3 John Walls Renal Unit, University Hospitals of Leicester NHS Trust, United Kingdom; 4 Department of Population Health Sciences, University of Leicester, Leicester, United Kingdom; 5 School of Sport, Exercise and Health Sciences, Loughborough University, Loughborough, United Kingdom; Medical University of Gdansk, POLAND

## Abstract

**Background:**

Cardiometabolic diseases are a major cause of morbidity and mortality in kidney transplant recipients (KTR) due to clustering of traditional and non-traditional risk factors including poor physical fitness and physical inactivity. Exercise may mitigate the risk of these diseases in this population but evidence is limited, and physical activity levels are low. The ECSERT randomised controlled trial assessed the feasibility of delivering a structured, home-based exercise intervention in KTR at increased cardiometabolic risk.

**Methods:**

Fifty KTR (>1-year post-transplant) were randomised 1:1 to: intervention (INT: a 12-week home-based combined aerobic and resistance exercise programme) or control (CTR: guideline-directed care). The *a-priori* thresholds for feasibility were: recruitment of 20% of eligible participants (≥2 participants per month); adherence (an average of ≥ 3 exercise sessions per week); and attrition (≤30%).

**Results:**

One hundred and seventy-one patients were screened and 94 (55%) were eligible and invited to take part in the study. Fifty of those invited (53%) were recruited across 22 months of recruitment. Consented participant characteristics were: age 50 ± 14 years (INT 49 ± 13; CTR 51 ± 15), 23 male (INT 10; CTR 13), eGFR 59 ± 19 ml/min/1.73m^2^ (INT 60 ± 20; CTR 61 ± 21), 35 White British (WB), 13 South Asian (SA), 1 Caribbean, and 1 Mixed ethnicity (INT 17 WB, 7 SA, 1 Mixed; CTR 18 WB, 6 SA, 1 Caribbean). Intervention participants (*n* = 22 completed) recorded an average of 4.4 ± 1.4 exercise sessions per week (aerobic 2.8 ± 1.1; strength 1.6 ± 0.5). Three participants withdrew from the intervention group (1 COVID-19 infection, 1 recurrent urine infections unrelated to the trial, 1 time/family circumstances) and one from the control group (lost to follow-up; 8% attrition). There were no serious adverse events reported.

**Conclusion:**

Despite previous evidence showing physical fitness and activity levels are low in KTR, the present results support that a structured, home-based exercise programme is feasible in this population. Specifically, *a-priory* recruitment, adherence, and retention thresholds were all exceeded. The groups were well matched and there was encouraging representation of female participants and participants from a non-white background. Thus, this study supports further development and testing of home-based programmes of exercise and activity for KTR.

**Trial registration:**

ClinicalTrials.gov NCT04123951

## Background

Kidney transplantation is the preferred modality of kidney replacement therapy for many patients with end-stage kidney disease (ESKD), offering survival advantages over dialysis therapy [1]. In kidney transplant recipients (KTR) cardiovascular disease (CVD) is still a leading cause of morbidity, mortality, and graft loss [2–4] and associates with traditional cardiometabolic risk factors [3,5,6], which drive classical atheromatous coronary artery disease, and non-traditional risk factors (e.g. endothelial dysfunction, systemic inflammation, acute rejection, anaemia, and deranged bone-mineral metabolism [7–9]) resulting in pathological changes in cardiovascular structure and function that are associatde with mortality [10]. Immunosuppressive agents are well known to worsen traditional [3] and non-traditional cardiometabolic risk profiles [11,12]. Generic CVD risk-stratification tools underestimate cardiovascular risk in chronic kidney disease (CKD) [8,13–15]; coronary revascularisation does not improve outcomes for KTR as it does in the general population [9] and cardiac events are more likely to be fatal [16].

Numerous epidemiological studies have demonstrated the association between low levels of physical activity and increased prevalence of CVD risk factors [17–19], and an inverse relationship between physical activity levels and both all-cause and CVD mortality [20,21]. Physical activity levels in KTR are low, with research estimating that the proportion of KTR reaching World Health Organisation recommendations is 27-52% [22–25].Whilst physical activity levels improve in the year following transplantation, they plateau after one year [23]. In the general population, lifestyle changes that increase physical activity through structured exercise lower mortality [26,27]. Despite this evidence, there is a lack of rigorous research into the role of increased physical activity and exercise in mitigating cardiovascular risk in KTR [28–30].

Whilst supervised exercise interventions in KTR improve cardiorespiratory fitness and a variety of traditional and non-traditional risk factors for CVD [29], they are not realistically deliverable in the current financial climate and have not translated to clinical practice. The deliverability of supervised programmes was further challenged by the COVID-19 pandemic, particularly in clinically vulnerable populations. Furthermore, exercise habits following in-centre supervised programmes are not maintained [31–33] which is potentially attributable to low levels of patient activation and a failure for such programmes to engender sustained lifestyle changes [34,35]. Given some of the barriers to physical activity and exercise expressed by KTR include lack of facilities, cost, time, access, and self-confidence, home-based programmes seem a plausible solution to address these issues. However, it cannot be assumed that such programmes will be acceptable to KTR, whose home-lives, social, and occupational circumstances are significantly different to dialysis and cardiac patients. Many KTR have had enforced sedentary lifestyles prior to transplantation as dialysis patients and their goals for rehabilitation as well as the disease processes at work may be different [36,37].

The ECSERT trial aimed to assess the feasibility, acceptability, and deliverability of a 12-week home-based programme of exercise and the trial procedures in this patient population.

## Materials and methods

### Trial design and participants

ECSERT (a pilot randomised controlled trial of a structured, home-based Exercise programme on Cardiovascular StructurE and function in Renal Transplant recipients) was a prospective, randomised, open-label, blinded endpoint (PROBE) feasibility study performed at one renal unit in the United Kingdom and all participants provided written informed consent. The full trial protocol is as previously published [38]. Stable adult KTR > 1-year post-transplantation were eligible to participate (Table 1). Reporting was informed by the Consolidated Standards of Reporting Trials (CONSORT) extension for randomised pilot and feasibility trials [39].

**Table 1:** ECSERT inclusion and exclusion criteria.

Inclusion criteria	Exclusion criteria
Prevalent KTR > 1 year Male or female, aged > 18 years old Willing and able to give informed consent for participation in the study Increased cardio-metabolic risk with at least one of: Diabetes mellitus Dyslipidaemia Hypertension Obesity (BMI > 30) History of ischaemic heart disease/cerebrovascular disease	Inability to give informed consent or comply with testing and exercise protocol for any reason Unable to undergo CMR scanning (incompatible implants, claustrophobia, allergy to agents etc.) Female participants who are pregnant, lactating, or planning pregnancy during the trial Scheduled elective surgery or other procedures requiring general anaesthesia during the trial Any other significant disease or disorder *

*i.e., significant co-morbidity including unstable hypertension, potentially lethal arrhythmia, myocardial infarction within 6 months, unstable angina, active liver disease, uncontrolled diabetes mellitus (HbA1c ≥  9%), advanced cerebral or peripheral vascular disease which, in the opinion of the patient’s own clinician, may either put the patient at risk because of participation in the trial, or may influence the result of the trial, or the patient’s ability to participate in the trial.

Abbreviations: BMI, body mass index; CMR, cardiac magnetic resonance; KTR, kidney transplant recipient.

### Ethics and governance

University of Leicester were the sponsor for this study (UOL 0714). The protocol was reviewed by the East Midlands-Nottingham 2 research ethics committee and was given a favourable opinion (REC ref 19/EM/0209) on 14/10/2019. Health Research Authority regulatory approval was given on 14/10/2019, and the study was adopted on the National Institute for Health Research (NIHR) portfolio on 26/09/2019. Local governance approval was granted by University Hospitals of Leicester (UHL) R&I on 31/01/2020. This study was prospectively registered with ClinicalTrials.gov (NCT04123951; 11.10.2019).

### Randomisation

Following baseline assessment, participants were randomly allocated (1:1) to either; (1) a 12-week home-based combined aerobic and resistance exercise programme (n  =  25) or; (2) control (n  =  25; receiving guideline-directed care). Randomisation was blocked (using computer-generated random permuted blocks with allocation concealment; https://www.sealedenvelope.com/simple-randomiser/v1/) to ensure periodic balancing. Given the nature of the intervention, it was not possible for participants to be blinded to their allocation or assessments. REB completed the randomisations.

### Intervention and comparator arms

#### Intervention group: 12-week home-based combined aerobic and resistance training programme.

The 12-week, home-based, structured exercise programme included aerobic and resistance training (4–5 sessions in total per week). Participants were advised to complete a warm-up and cool-down prior to and following each session, respectively. Participants continued to receive usual clinical care.

#### Aerobic component.

The aerobic component of the programme was walking, jogging, cycling, or similar, depending on resources available and participant preference. Participants were asked to complete 2–3 sessions per week using a rating of perceived of exertion (RPE [40]) of 13–15 (somewhat hard-hard) for 20–30 min.

#### Resistance component.

The resistance component of the exercise programme included a combination of 6-8 exercises per session chosen by the participant from a pool of twelve exercises (to provide variety) targeting upper and lower body and core muscle groups, using free weights and/or resistance bands. The chosen pool of exercises included: squat, hip abduction, lunge, calf-raise, side-lunge, bicep-curl, bent-over row, reverse-fly, lateral-raise, chest-press, side-bends, and standing trunk rotation. Participants were asked to complete 6-8 resistance exercises twice a week (but not on consecutive days to allow appropriate recovery). Initially they were advised to complete 1–2 sets of 10 repetitions (at 60% 1-repetition maximum [RM]), gradually increasing to 3–6 sets of 10 repetitions with a minimum of 30 sec rest between sets with an aim of increasing muscle size. Whilst participants could do aerobic and resistance sessions on the same day if they wished, they were advised to have at least one day in between resistance sessions for recovery.

Participants were provided with an exercise diary which included additional instructions, dumbbells and resistance bands, and access to educational and instructional videos. Participants received a telephone call from a member of the research team every two weeks in order to discuss progression of the exercise and address any issues.

#### Control group: ‘Guideline-directed care’.

Participants in the control group were asked to maintain their current lifestyle and exercise habits throughout the study. This included continuing to attend any scheduled clinic appointments and taking prescribed medication as normal. As part of routine care, KTR are recommended to take regular exercise and maintain a healthy lifestyle [41]. This advice was reiterated to patients in the control group to ensure the intervention was being appropriately compared to best-practice guideline-directed care.

### Study timeline

#### Baseline assessments.

Baseline assessments were carried out prior to randomisation and where possible on the same day and in conjunction with routine clinical appointments to prevent additional travel. These assessments included: baseline and clinical characteristics, cardiopulmonary exercise testing (CPET), blood and urine sampling, habitual physical activity (GENEActiv accelerometry), strength testing (isokinetic dynamometry and handgrip), body composition, physical function (timed up and go [TUAG], sit to stand 60 [STS60], gait speed], postural stability, and a series of questionnaires (symptoms, sleep, fatigue, quality of life, patient activation, physical activity, and health literacy).

#### Follow-up assessments.

Final assessments were conducted for the exercise and control groups within 7 days of completing the 12-week exercise or control period. Assessments completed were identical to the baseline visit with the addition of a ‘patient satisfaction questionnaire’. Participants in the intervention group were invited to take part in a qualitative semi-structured intervention to explore experiences of participating (data to be presented elsewhere).

### Data collection and management

Data from all time points was collected in case report forms (CRFs) by the trial team. All data was entered into a secure database only accessible on password-protected computers at UHL and University of Leicester by relevant members of the study team. No identifying information was kept in electronic form. All source data and original participant identities were kept in a locked office in the trial site file only at UHL.

### Data analysis

The *a priori* thresholds for key specific feasibility and acceptability criteria were chosen by the lead researchers based on previous research and patient and public involvement/engagement (PPIE) events in this population and/or at this centre [42,43]. Achievement of these thresholds combined with consideration of other secondary feasibility outcomes would indicate that the trial could progress to a fully powered randomised controlled trial (RCT) and identify areas of trial design or conduct that would require adjustment, or particular adaptations that would be needed to the intervention [44]. Data on eligibility, recruitment, and attrition were summarised using descriptive statistics and confidence intervals where applicable, and the flow of participants was presented in a CONSORT diagram. Data are presented as complete case analyses and as mean ±  SD or median (IQR) where data is not normally distributed.

### Feasibility outcomes

Table 2 outlines the key feasibility outcomes and criteria. It also outlines additional feasibility outcomes to aid the development of future studies.

**Table 2:** Feasibility outcomes.

	Definition	Criteria
**Key criteria**		
Recruitment rate	The percentage of patients eligible and invited who consented to the trial and the monthly recruitment rate. Calculated by the number of participants consented/number eligible and invited x 100%.	Success of 20% of eligible subjects and ≥ 2 participants per month.
Adherence to the exercise programme	The number of completed sessions per week. Calculated from the self-reported data from the exercise diaries as the average number of sessions reported per week across the 12 weeks by all participants (not taking into account intensity, duration, or quantity performed).	Report completion of an average ≥ 3 exercise sessions per week.
Attrition rate	The percentage of participants that dropped out of the study. Calculated by the number of participants withdrawn/number completed follow-up assessments x 100%.	≤30%
Other feasibility outcomes*		
Eligibility	The percentage of patients screened who were eligible. Calculated by number of eligible patients/number screened for eligibility x 100%.	–
Acceptability of randomisation	Comparison of the final baseline characteristics and identification of any stratification variables, if applicable. Difference tests were not performed as per CONSORT recommendations [45,46].	–
Compliance with the exercise programme	Self-reported exercise session intensity, duration (for aerobic), and numbers of exercises performed (for resistance) from diary entries.	–
Outcome acceptability	The number and percentage of missing data for each outcome measure. Data were classified as missing for questionnaire measures where there was insufficient data to calculate meaningful scores. Data on accelerometery was considered missing if less than four days of wear-time were recorded [47].	–
Safety and adverse events	The number of self-reported injuries or adverse events throughout the trial. Including reasons for missed exercise sessions.	–
Direct patient feedback	An anonymous patient satisfaction questionnaire (Likert scaling) was given to each participant in the intervention group who completed the follow-up assessments. This was split into question groups: general, exercise programme, assessments, and overall satisfaction. The mean response to each question was reported.	–

*To supplement the key criteria to inform future studies.

## Results

### Recruitment and allocation

**Fig 1:**
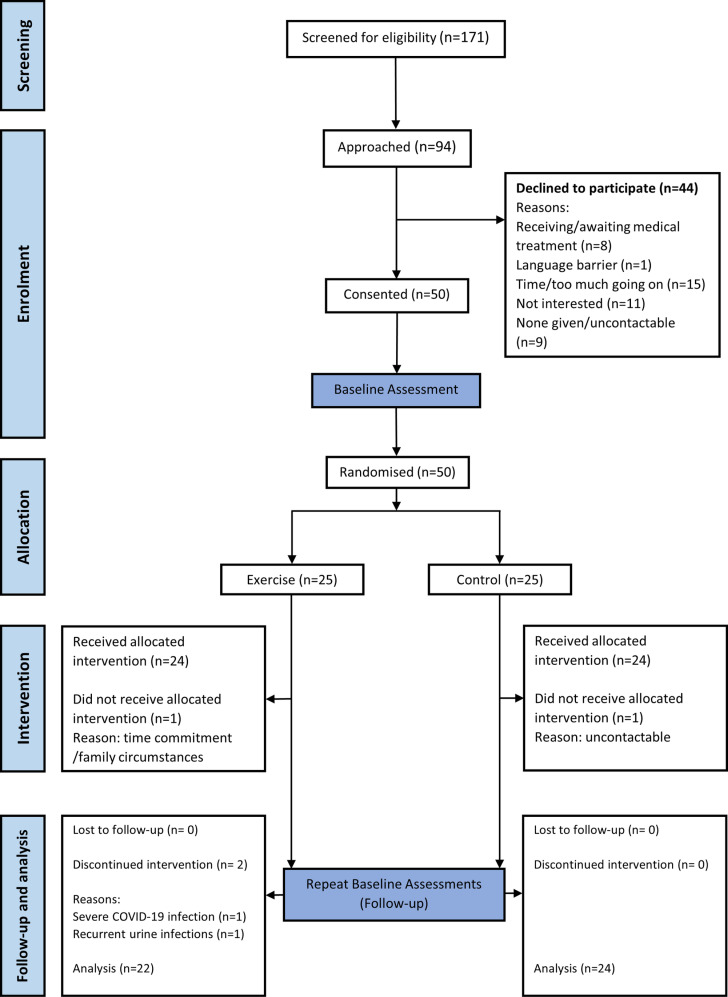
CONSORT diagram for the flow of participants through the ECSERT trial.

Participants were recruited between 9th March 2020 and 23rd February 2023. Recruitment was ended due to achieving the planned sample size. The study was paused due to COVID-19 between late March 2020 and October 2020 (recruitment recommenced in November 2020). There were four further inactive recruitment months due to staff ill-health. In total there were 23 months of active recruitment. Fig 1 presents the flow of participants through the trial.

One hundred and seventy-one patients were screened for eligibility and 94 met the recruitment criteria for the trial (55%, 95%CI: 48 to 62). Ninety-four patients were approached, of which 50 (53%, 95%CI: 43 to 63) were recruited and consented into the trial. Of those who offered a reason for declining participation in the trial, not being interested, lack of time, or having too much going on, and receiving or awaiting medical treatment for co-morbidities were the most commonly stated reasons for non-participation. The mean age of patients who declined to participate was 54 ± 13 years and 23 were male (52%). Fifty participants (100%) completed the baseline assessments and none withdrew prior to randomisation.

### Baseline demographics and randomisation acceptability

Table 3 presents the participant and clinical characteristics at baseline. Baseline values for secondary outcomes are provided within supplementary material (S1–S8 Table). Participants were 50 ± 14 years old with an average eGFR of 61 ± 20 ml/min/1.73m^2^. Sixteen participants (32%) were of a non-White British background and 27 (54%) were female. The groups were well-matched across all parameters apart from body mass. There was a 5.8% difference in body mass between the intervention and control groups.

**Table 3:** ECSERT baseline demographics and clinical characteristics.

	Intervention (n = 25)	Control (n = 25)	All (n = 50)
Age (years)	49 ± 13	51 ± 15	50 ± 14
Sex, n (%)			
Male	10 (40)	13 (52)	23 (46)
Female	15 (60)	12 (48)	27 (54)
Ethnicity, n (%)			
White/White British	16 (64)	18 (72)	34 (68)
South Asian	8 (32)	6 (24)	14 (28)
Caribbean	0 (0)	1 (4)	1 (2)
Other ethnic	1 (4)	0 (0)	1 (2)
KRT (months)^a^	46 (20-107)	46 (26-165)	46 (25-115)
eGFR (ml/min/1.73 m^2^)	60 ± 20	61 ± 21	61 ± 20
Body mass (kg)	84 ± 26	80 ± 14	82 ± 21
Smoking status, n (%)			
Current	3 (12)	1 (4)	4 (8)
Previous	5 (20)	11 (44)	16 (32)
Co-morbidities, n (%)			
Hypertension	24 (96)	23 (92)	47 (94)
Type II Diabetes	5 (20)	4 (16)	9 (18)
Heart condition	0 (0)	1 (4)	1 (2)
Hyperlipidaemia	14 (56)	14 (56)	28 (56)
Medication, n (%)			
CNI	25 (96)	25 (100)	49 (98)
mTOR inhibitor	1 (4)	0 (0)	1 (2)
Mycophenolic acid	24 (96)	21 (84)	45 (90)
Steroid	19 (76)	19 (76)	38 (76)
Antihypertensive	22 (88)	22 (88)	44 (88)
Diabetes therapy	5 (20)	4 (16)	9 (18)
Statin	12 (48)	11 (44)	23 (46)
Vitamin D/Calcium	12 (48)	14 (56)	26 (52)

Notes: Unless otherwise indicated, values for categorical variables are expressed as integer (% of n); values for continuous variables as mean ±  SD. ^a^median (IQR).

Abbreviations: CNI, calcineurin inhibitor; eGFR, estimated glomerular filtration rate; KRT, kidney replacement therapy; mTOR, mammalian target of rapamycin.

### Attrition rate

Forty-six participants (92%, 95%CI: 85 to 99) who completed baseline assessments were retained and completed follow-up assessments (attrition 8%, 95%CI: 1 to 16). Two participants withdrew from the trial prior to the 12-week intervention commencing (1 intervention due to time/family circumstances and 1 control lost to follow-up). Two participants from the intervention group withdrew during the 12-week programme; one due to severe COVID-19 infection and one due to recurrent urinary tract infections unrelated to the trial.

### Exercise programme

#### Number of sessions completed (adherence).

**Fig 2:**
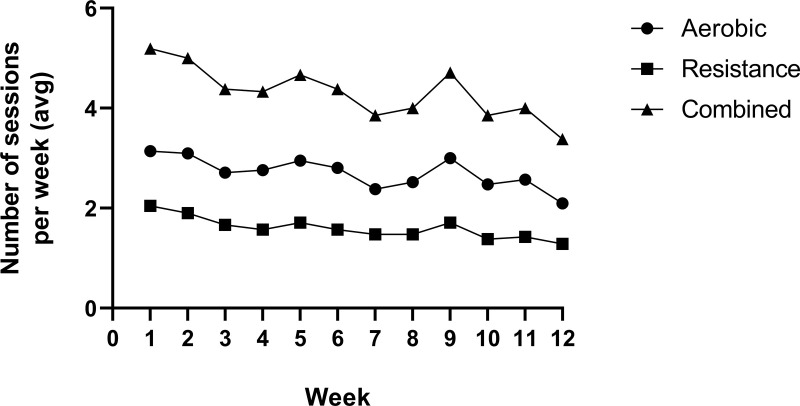
Average number of sessions reported each week over the 12-week programme.

Of the 22 participants in the intervention group who completed the follow-up assessments, 21 returned their 12-week exercise diaries. Participants recorded an average of 4.3 ± 1.5 exercise sessions per week across the 12 weeks. This was an average of 2.7 ± 1.3 aerobic sessions and 1.6 ± 0.5 resistance sessions per week. Fig 2 shows the average number of sessions completed each week. The average total number of sessions completed over the 12 weeks was 51.8 ± 17.4. The minimum total number of sessions participants were asked to complete over the 12 weeks was 48 and the maximum was 60 meaning that adherence rates were over 100% for the minimum target and 86% for the maximum target.

#### Aerobic component (compliance).

Walking and cycling were the most common self-reported exercise type (supplementary material, S1 Fig). The average RPE per session across the 12 weeks was 13 ± 1 (range 11-16). The average reported session duration was 48 ± 24 mins (range 5-300 mins).

#### Resistance component (compliance).

The top three performed resistance exercises were the bicep curl, calf raises, and the side bend (supplementary material, S2 Fig). For recorded sessions, the average number of exercises performed per session was 7.4 ± 0.8. The average number of sets of 10 repetitions over the 12 weeks was 2.8 ± 0.6 (1.8 ± 0.6 in week 1 and 3.5 ± 0.6 in week 12). Eighteen participants reported increasing the dumbbell weights across the 12 weeks (average increase of 1.5 kg). Example: bicep curl, the average weight used in week 1 was 2.5 kg and 4.4 kg in week 12.

#### Summary (adherence and compliance).

Table 4 provides a summary of the key exercise instructions compared to the average reported values by participants during the 12-week exercise programme.

**Table 4:** Summary of exercise instructions versus actual recorded averages.

	Instruction	Average recorded
**Aerobic**		
Sessions per week	2–3	2.7 ± 1.3
RPE per session	13–15	13 ± 1
Duration per session	20-30 min	48 ± 24 min
**Resistance**		
Sessions per week	2	1.6 ± 0.5 *
Number of exercises per session	6–8	7.4 ± 0.8
Number of sets of 10	1-2 increasing to 3-6	1.8 ± 0.6 (Week 1) 3.5 ± 0.6 (Week 12)

Abbreviations: RPE, rating of perceived exertion.

* Nine participants recorded 2 sessions per week.

#### Outcome acceptability.

**Fig 3:**
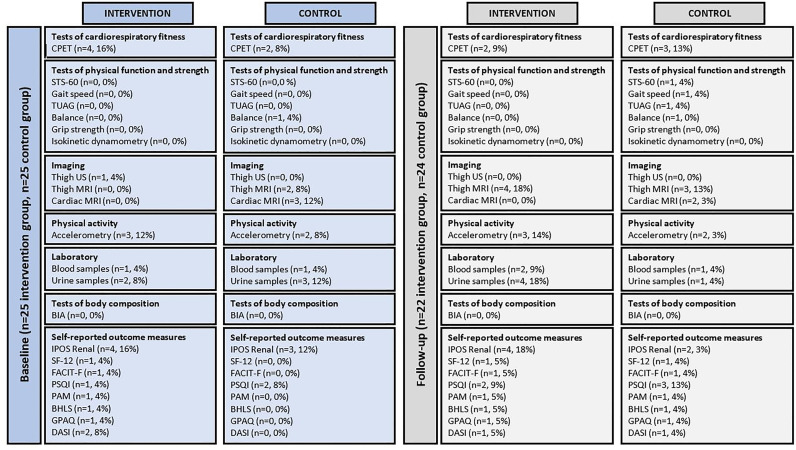
Missing data for each outcome measure. BHLS, brief health literacy screen; BIA, body mass index; CPET, cardiopulmonary exercise test; DASI, Duke activity status index; FACIT-F, Functional Assessment of Chronic Illness Therapy-Fatigue; GPAQ, Global Physical Activity Questionnaire; IPOS-Renal, Integrated Palliative Outcome Scale; MRI, magnetic resonance imaging; PAM, patient activation measure; PSQI, Pittsburgh Sleep Quality Index; SF-12, 12-Item Short Form Health Survey; STS-60, sit to stand 60; TUAG, timed up and go; US, ultrasound.

A summary of missing data is provided in Fig 3. Completion rates for all outcome measures were high at baseline and follow-up. The largest missing data was in the CPET. Some participants were unable to manage the intensity of the test, however, this was still a small number. Two of these tests were missed due to atmospheric temperature being too high to safely perform the test as air conditioning was not permitted due to local protocols during the COVID-19 pandemic. Tests of physical function and strength were the most complete.

### Safety, adverse events, and reasons for missed exercise sessions

Overall, there were no serious adverse events reported relating to the trial, the trial procedures, or the exercise programme. One participant reported a pulled thigh muscle whilst running but this was resolved within two weeks. All other reports of illness and injury were unrelated to the trial.

In the intervention group, eight participants reported holidays of varied lengths during the 12-week intervention period. Most participants were able to maintain aerobic exercise but not resistance training due to lack of equipment whilst away. Eight participants reported cold or flu or COVID-19 infection at various points during the programme. Three participants reported urinary tract infections. Two participants recorded hospital stays (one for deep vein thrombosis and one for repeated infections). Five participants had pain relating to pre-existing musculoskeletal conditions or injuries which impacted the type of exercise that they could perform. One participant reported mental health as a barrier to completing some exercise sessions. One participant developed acute graft rejection during follow-up assessments, unrelated to the trial.

In the control group, five participants reported cold or flu symptoms at various points during the 12-week trial period. Two participants reported urinary tract infections, one resulted in hospital admission. Four participants had pain relating to pre-existing musculoskeletal conditions or injuries. One participant reported panic attacks and migraines.

### Participant feedback

**Fig 4:**
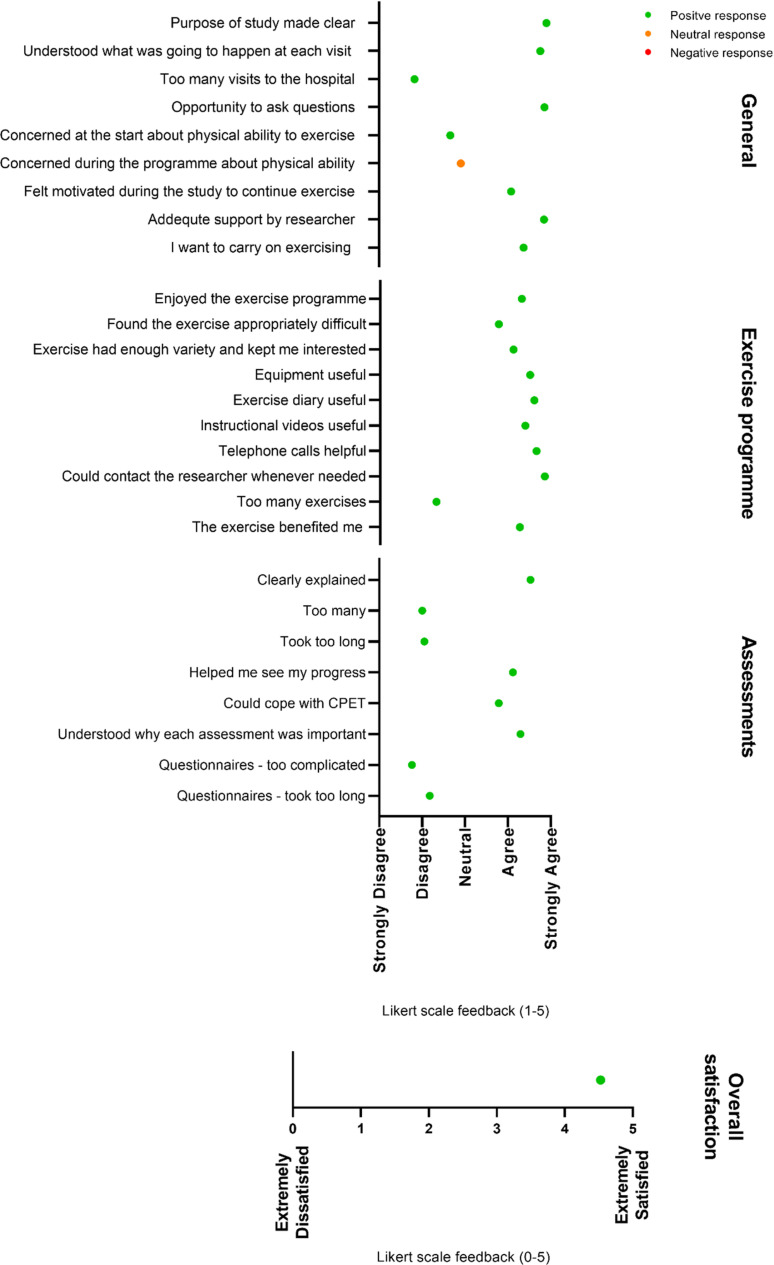
Feedback questionnaire responses from the intervention group (n=  21). Note: Dots represent mean response.

**Fig 5:**
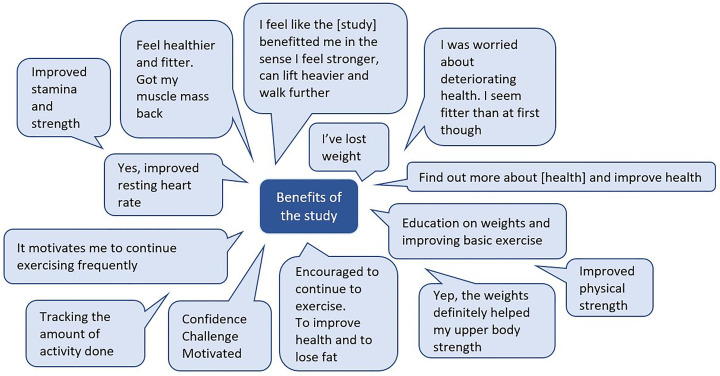
Free-text responses on the benefits of the study.

Twenty-one participants who completed the exercise programme returned the anonymous participant satisfaction questionnaires. A summary of mean scores per question is provided in Fig 4. For the general questions, participants scored positively on all, apart from one neutral response around concern during the programme about physical ability. Participants felt that the study purpose was made clear, there were not too many visits, and they had the opportunity to ask questions. They felt motivated to exercise during the study, felt adequately supported, and wanted to continue exercising after the study. For the exercise programme-specific questions, all responses were positive. Participants reported enjoying the programme, that it was adequately difficult, it was varied enough to maintain interest, and that it benefited them. Participants found the exercise equipment, instructional videos, and telephone calls useful or helpful. For the assessments, participants scored all aspects positively. They reported that assessments were clearly explained and that they understood the importance. There were not too many assessments and they were not too long. Participants reported that the assessments helped them see their progress. Some participants reported buying their own dumbbells to continue exercise after the programme. Overall satisfaction with the study was very positive. Fig 5 provides a summary of the free-text responses answering whether participants felt like the study had benefitted them in any way. All responses were positive.

Three participants responded to the question about disappointment in the programme. One participant was disappointed in their personal willpower. One participant reported that the study did not take into account certain health implications, specifically having a fistula. They felt worried about lifting weights with the fistula arm. One participant was frustrated that they pulled their muscle and felt it was because they did not warm up correctly. Eight participants made recommendations on what could be done to improve the experience of the exercise programme. One participant would have liked longer tuition time to increase familiarity with the exercises, and a longer programme overall, and one participant would have liked group sessions or one-to-one sessions. Three participants would have liked changes to the exercises; more exercises without weights, more leg exercises, and the addition of structure and specific mobility exercises. One participant suggested having an app to track progress. One participant would have liked more scheduled exercise and one participant would have liked more target and goal setting, personal to them. S9 Table (supplementary material) provides a summary of factors that participants recorded as the hardest and the most enjoyable aspects of the programme.

## Discussion

This study showed that against the three key *a priori* thresholds (recruitment, exercise programme adherence, and attrition) a trial of a home-based exercise programme is feasible in the stable KTR population. This is strengthened by the review of the additional feasibility measures (eligibility, compliance with the exercise programme, outcome acceptability, and safety) which yielded positive findings. These findings are supported by positive feedback directly from the participants.

### Eligibility and recruitment

Within the study, 55% of patients screened were eligible to take part and 53% of those who were eligible were recruited and consented. This is greater than in other trials of exercise programmes in KTR, although not solely home-based [48]. De Smet and Van Craenenbroeck [48] reported 35% of potential participants were eligible and recruited in a pooled review and analysis of 11 exercise studies (n = 562/1621). In our previous work, 60% of patients screened (KTR) were eligible to take part but only 23% were recruited [42]. However, this was a three-times-per-week supervised high-intensity interval training intervention, both factors which may have influenced the decision to take part. In the two other trials of solely home-based exercise in KTR, recruitment rates were 54% [49] and 70% [50] making the present study comparable and potentially supporting the hypothesis that home-based exercise may be preferred to address the lack of time barrier to exercise commonly cited by KTR [51]. This is supported by the resistance training study of Karelis et al. [52] who amended their protocol from three supervised exercise sessions per week to one supervised session and two home-based sessions after experiencing recruitment challenges. Their recruitment rate was 47% of eligible participants which is again comparable with the present study.

De Smet and Van Craenenbroeck [48] noted that exclusion criteria for exercise studies in KTR are unsurprisingly strict which limits the reach (the extent to which the target audience comes into contact with the programme or intervention) and generalisability of trial results. This is particularly prominent in trials of an unsupervised nature, such as those involving home-based exercise. Despite this, the present study reached an acceptable level of eligibility. The present trial also excluded patients who were < 1-year post-transplant due to the turbulent nature of reaching transplant stability and factors such as increased infections and rejection episodes [53–55]. This is reflected in data which suggests that physical activity levels immediately drop one-month post-transplant, then gradually increase, and plateau after one year [22]. Therefore, it was not considered the most beneficial time to test an intervention which influences levels of physical activity and aims to engender a sustainable change whilst they were already naturally changing. For these reasons, trials moving forward are unlikely to be able to improve the eligibility levels further unless specifically implementing interventions to improve initial post-transplant mobility.

The age and sex of those patients who were eligible but declined to participate in the trial were similar to those of participants within the trial. This is positive, as it suggests that reasons for declining were not related to participants’ age, sex, or other protected characteristics making the intervention or trial inappropriate. One of the most common reasons for declining to participate was lack of time or having too much going on. This was also seen in one of our previous exercise trials in KTR [42] but to a lesser extent in the present study which could be due to the home-based nature of the exercise programme. In comparison to the prevalent KTR population within the UK, the current trial has a lower median age (57 v 50 years), a lower percentage of males (61 v 46%), and a lower percentage of white participants (78 v 68%) [56] which may limit the external validity. However, for the Leicester population, the percentage of male KTR (58%) and those of white ethnicity (73%) are comparable [56], highlighting the importance of multi-centre trials. It is also of note that Leicester is a highly active centre for kidney research. This engagement and openness to research is likely to have a positive impact on trial recruitment and we therefore cannot generalise to other kidney units within the UK. Similarly, as with many exercise trials, selection bias is likely. Patients who are more motivated to engage in physical activity are likely to face fewer barriers and be ‘willing and able’ to provide informed consent as per criteria. Interpretation of findings should take this into account.

### Baseline demographics and outcome acceptability

Baseline demographics were well-matched across both groups which is encouraging for future RCTs. There was a 5.83% difference in weight between the intervention and control group which could affect efficacy results. A reduction in weight of ≥ 5% is considered clinically relevant [57,58] for the clinically significant improvement in factors such as CVD risk, diabetes mellitus, lipidaemia, osteoarthritis, and health-related quality of life [57]. This must be taken into account when conducting statistical analysis and interpreting results of future trials.

Outcome acceptability and missing data are inconsistently reported in the literature. It is important to establish outcomes that may not be appropriate for future definitive trials as missing data can lead to systematic differences between study groups, introduce risk of bias, reduce statistical power, and make interpretation and generalisation of the results difficult [59,60]. In the present study, missing data was limited and was largely due to equipment or situational reasons. Therefore, this set of outcome measures could be used successfully in future trials. The outcome measures align with some of the core outcomes (cardiovascular disease, life participation, and graft heath) and many from the other tiers of the Standardised Outcomes in Nephrology (SONG) initiative for transplant trials (SONG-Tx) [61].

### Attrition rate

There was an overall attrition rate of 8% (12% in the intervention group [n  =  3] and 4% in control [n  =  1]) which was below the key criteria limit (≤30%). Twelve out of the 17 exercise training RCTs reviewed by De Smet and Van Craenenbroeck [48] reported attrition rates and these showed higher overall attrition in the intervention and control groups than in the present study (26% versus 25%, respectively). Attrition in the study of 167 KTR by Painter et al. [49] was very high (42%; 49% control versus 35% exercise). There were two key factors which may have influenced this difference with the present study. First, the home-based intervention was for one year, giving four times longer for participants to withdraw. However, 73% withdrew between baseline and six months. Secondly, they recruited participants within two months post kidney transplantation. The home-based exercise trial by Michou et al. [50] was more comparable to the present study (28 KTR randomised to a 6-month exercise intervention or ‘usual lifestyle’ control). They reported an attrition rate of 25% (22% exercise and 29% control) which is still higher than the present study. Given the changes to the present trial protocol due to COVID-19 (38), it is possible that attrition was lower due to decreased patient burden of taking part in the trial (research visits were combined with clinical appointments and less work and social commitments due to shielding). Future trials may consider the combination of clinical appointments and visits where feasible to limit patient burden.

### Adherence to and compliance with the exercise programme

Participants reported an average of 4.3 exercise sessions per week over 12 weeks, achieving the pre-defined adherence criteria of three sessions per week. In the review of home-based exercise interventions in CKD, Pedroso et al. [62] found that eight out of 14 studies reported adherence and the percentage of sessions performed ranged from 57% to 83%. In the exercise studies in KTR (not home-based), eight of the 17 RCTs reported adherence and all but one study reported rates of over 80% [48]. The present study also reported session adherence rates (average total number of sessions performed over the 12 weeks) of over 80%. In the two studies of home-based exercise interventions in KTR, Michou et al. [50] did not explicitly report intervention adherence and Painter et al. [49] reported classification of active and inactive at 12 months (67% in the exercise group and 36% in the control group). The shorter duration of the current study may have positively influenced the adherence to the programme, especially as Painter et al. [49] also employed fortnightly telephone calls to provide support and monitor progress. This is supported by this study which showed that the number of sessions completed very slowly declined over the 12-week programme. This is not uncommon, particularly in studies of a home-based nature [63–65], but future trials of longer interventions and follow-ups should seek to identify factors or programme features which encourage sustained participation.

When looking into the details of the individual components of exercise, participants recorded 2.7 ± 1.3 aerobic sessions and 1.6 ± 0.5 resistance sessions. We asked participants to perform 2-3 aerobic sessions and 2 resistance sessions per week. Although participants did not quite reach an average of 2 resistance training sessions per week, nine out of the 22 participants did achieve this. Others were unable to perform some of the sessions due to holidays and the difficulty of needing equipment there to complete them. However, some participants reported positive enjoyment and tangible results (from written feedback) for the resistance training and some reported buying their own equipment to maintain training after the study had ended. The two resistance training sessions per week recommendation comes from the UK Chief Medical Officers guidance and that of the recent UK Clinical Practice Guidelines for Exercise and Lifestyle in Chronic Kidney Disease [66,67]. Resistance training has been associated with reduced mortality in the general population [68] as has increased levels of strength [69,70]. The same is true for KTR, where low muscle strength associates with mortality [71] and increased hospitalisations [72]. With this in mind, any increase in resistance training which induces improvements in muscle strength is beneficial.

Participants reported on average meeting the intensity targets for the aerobic component of the programme. However, this was at the lower end of the target (13-15 RPE) and the most popular activity reported by far was walking. This is likely due to the ease and not requiring any equipment. A RPE within this range is considered moderate intensity, but, participants did not meet the UK Chief Medical Officers guidance or the UK Clinical Practice Guidelines for Exercise and Lifestyle in Chronic Kidney Disease of 150 min of moderate intensity exercise per week [66,67]. We did not use the guidelines as an aim for this project as PPIE feedback suggested that these were overwhelming for patients. The aim was to get KTR active in any way and future trials with longer intervention periods should seek to build up to achieving these guidelines. However, there is an abundance of literature showing the value of even small increases in daily steps on mortality and health [73–75]. The only self-reported feedback that was neutral and not positive was the statement pertaining to having concern during the programme about physical ability. This could indicate that participants were afraid to push themselves which is supported by the study from Zelle et al. [76] who reported fear of movement negatively correlated with physical activity levels in 487 KTR. Although not possible in the present study due to COVID-19, in-person instructional sessions and the option for some supervised sessions may help to build self-confidence and self-efficacy which also negatively correlated with fear of movement [76]. The need for some initial supervised support in becoming physically active was identified in earlier qualitative work, as was the need to discover personal limits in how far KTR can push themselves during exercise [51].

A limitation to the study is the use of self-reported data on exercise adherence and compliance. We cannot be certain that participants correctly completed the exercise diaries, and indeed self-reported levels of physical activity are known to be overestimated [77]. The exercise diary was reviewed by a PPIE group to ensure it was appropriate and the initial instructional session involved training on how to complete the diary with the aim to mitigate some of the limitations. The use of a physical diary is accessible, low cost to administer, but resource intensive to evaluate. Future trials may explore the use of wearable technologies; the use of which has been evaluated in the context of oncology trials and clinical practice identifying the uses and challenges [78]. In kidney care, they have been highlighted as long-term monitoring tools and to provide a positive stimulus to increase physical activity levels [79]. Consideration must be given to the impact of wearing an activity tracker; studies have shown that improvements in activity can come from wearing a tracker alone [80], making it difficult to attribute the cause of change.

### Safety and adverse events

Only one minor adverse event was reported during the study (pulled thigh muscle during running) which was self-diagnosed by the participant as being due to not adequately warming up for the session as per instructions. This is in line with the conclusions of both recent reviews of home-based exercise trials in patients living with CKD stating that home-based exercise is safe and feasible [62,81]. This is similar to the conclusion of De Smet and Van Craenenbroeck [48] who also noted that this is likely due to studies’ criteria of stable graft function and absence of severe comorbidities.

A high number of sessions were missed due to ill health or musculoskeletal pain. Similarly high levels of cold and/or flu symptoms and urinary tract infections were reported in our previous in-centre exercise trial for KTR [42]. Reports in the intervention group were not dissimilar to the control group. This is not unexpected as KTR are susceptible to an array of viral pathogens due to immunosuppression [82,83]. This is a relevant finding for the design of a future trial as periods of acute ill health limit the ability to engage with the programme with continuity. Furthermore, deconditioning occurs following acute illness and a future trial may need to consider extending the exercise intervention period for those afflicted by significant periods of intermittent illness whilst engaging in the exercise programme. Specific advice, supervised sessions, or ‘return to exercise’ guidance may be needed to help the return to full exercise. Abnormalities of the structure and function of the musculoskeletal system are also common post-transplant and occur potentially as a result of abnormalities in bone and mineral metabolism likely due to steroid therapy and other medications [84]. These factors are largely unavoidable; however, care should continue to be taken in future studies to provide adaptable and varied exercise programmes to avoid discomfort whilst exercising. Although evidence is limited, one study showed improved bone mineral density with combined aerobic and resistance training compared to control [85] therefore exercise in a controlled manner should still be encouraged in these patients.

## Conclusion

In summary, the results of the trial suggest that a trial of home-based aerobic and resistance training in KTR is feasible, safe, and positively received. The three key pre-defined criteria (recruitment, exercise programme adherence, and attrition) were achieved. And further additional feasibility criteria (eligibility, compliance with the exercise programme, outcome acceptability, and safety) yielded positive results. Small changes to the programme may be considered in future trials and/or physical activity programmes that address the small drop-off in session adherence across the time period and address the concerns around physical ability to complete the exercise. This may be in the form of in-person instructional sessions, the option for some supervised training if desired, and the use of wearable technologies to monitor activity. Overall, these results provide important information for the development of future trials and programmes of home-based exercise in KTR.

## Supporting information

S1 File ECSERT feasibility supplementary material file. (DOCX)

S1 Data Raw data. (XLSX)
