# Supplementary material for: A structured, home-based exercise programme in kidney transplant recipients (ECSERT): A randomised controlled feasibility study
Source: PLoS One. 2025 Feb 24;20(2):e0316031. doi: 10.1371/journal.pone.0316031 (PMC11849866; doi:10.1371/journal.pone.0316031)
Supplement: S1 File — (DOCX) [file pone.0316031.s001.docx]

**S1 File: ECSERT feasibility supplementary material file**

**Supplementary materials**

Table S1. Summary of cardiorespiratory fitness values at baseline

| **Variable** | **n** | **Baseline (Mean ± SD) (Intervention)** | **n** | **Baseline (Mean ± SD) (Control)** |
| --- | --- | --- | --- | --- |
| Resting |  |  |  |  |
| SBP (mmHg) | 22 | 133.59±20.78 | 24 | 130.33±19.99 |
| DBP (mmHg) | 22 | 79.23±12.83 | 24 | 74.81±9.52 |
| Gas Exchange Threshold |  |  |  |  |
| V̇O_2_ (mL/kg/min) | 18 | 12.22±3.00 | 19 | 12.47±2.37 |
| V̇O_2_ (L/min) | 18 | 1.01±0.33 | 19 | 0.99±0.19 |
| V̇O_2peak_pred (%) | 18 | 48.72±16.04 | 19 | 51.77±12.71 |
| V̇CO_2_ (L/min) | 17 | 0.93±0.37 | 18 | 0.92±0.32 |
| V̇E (L/min) | 16 | 31.10±11.45 | 19 | 31.18±9.28 |
| Peak |  |  |  |  |
| V̇O_2peak_ (mL/kg/min) | 19 | 20.15±5.18 | 21 | 21.36±6.96 |
| V̇O_2peak_ (L/min) | 19 | 1.66±0.60 | 21 | 1.70±0.53 |
| V̇O_2peak_pred (%) | 19 | 80.06±21.25 | 19 | 87.20±23.89 |
| Max WR (watts) | 20 | 124.30±44.86 | 20 | 126.00±39.41 |
| Max HR (bpm) | 19 | 147.47±25.75 | 21 | 144.19±27.46 |
| V̇CO_2_ (L/min) | 18 | 1.88±0.63 | 19 | 1.59±0.48 |
| V̇E (L/min) | 19 | 68.56±25.43 | 21 | 72.94±22.07 |
| V̇E/V̇CO2 slope | 18 | 33.99±7.35 | 20 | 36.30±9.37 |
| V̇E/V̇O2 ratio | 18 | 38.26±6.25 | 19 | 37.58±5.21 |
| O_2_ pulse (ml/beat) | 15 | 10.8±3.61 | 12 | 12.3±2.91 |
| RO_2_ pulse (mL/kg/beat) | 15 | 0.13±0.03 | 12 | 0.15±0.32 |
| HR recovery (%) | 15 | 13.38±4.48 | 17 | 14.42±5.26 |
| HR recovery (bpm) | 15 | 20.27±7.89 | 17 | 21.94±9.34 |

Abbreviations: DBP, diastolic blood pressure; HR, heart rate; pred, predicted; R, relative; SBP, systolic blood pressure; V̇CO_2_, volume of expired carbon dioxide; V̇E, volume of expired air per minute; V̇O_2_, oxygen uptake; WR, work rate

Table S2. Summary of physical function, lower limb strength, and postural stability variables values at baseline

| **Variable** | **n** | **Baseline (Mean ± SD) (Intervention)** | **n** | **Baseline (Mean ± SD) (Control)** |
| --- | --- | --- | --- | --- |
| Physical function |  |  |  |  |
| STS-60 (reps) | 22 | 23.73±6.98 | 23 | 21.35±5.42 |
| Gait speed (m/s) | 22 | 1.08±0.18 | 23 | 1.05±0.15 |
| TUAG (s) | 22 | 8.67±1.77 | 23 | 9.60±1.85 |
| HGS (kg) | 21 | 30.41±9.93 | 24 | 28.31±9.70 |
| Postural stability |  |  |  |  |
| Velocity (mm/s) | 22 | 13.07±6.40 | 22 | 13.65±8.74 |
| COP (mm²) | 22 | 28.37±18.44 | 22 | 44.41±82.53 |
| Lower limb strength |  |  |  |  |
| Isometric Peak Torque (N·m) | 22 | 148.74±69.21 | 23 | 132.07±61.28 |
| Avg Peak Torque (N·m) | 22 | 137.58±66.06 | 23 | 120.71±59.64 |
| Avg PT/BW (%) | 22 | 163.68±51.16 | 23 | 154.53±78.17 |
| Isokinetic 60°s Peak Torque (N·m) | 22 | 116.25±44.87 | 23 | 101.50±43.52 |
| Isokinetic 60°s Avg Peak Torque (N·m) | 22 | 101.07±41.76 | 23 | 90.26±40.80 |
| Isokinetic 60°s Avg PT/BW (%) | 22 | 139.70±36.32 | 23 | 129.64±59.57 |
| Isokinetic 60°s Work/BW (%) | 22 | 162.86±53.11 | 23 | 140.78±63.42 |
| Isokinetic 60°s Avg Power (watts) | 22 | 66.28±34.24 | 23 | 55.35±27.89 |
| Isokinetic 60°s Total Work (J) | 22 | 561.28±256.58 | 23 | 459.36±206.55 |
| Isokinetic 120°s Peak Torque (N·m) | 21 | 93.64±35.70 | 23 | 75.82±31.91 |
| Isokinetic 120°s Avg PT/BW (%) | 21 | 112.08±30.04 | 23 | 96.86±42.43 |
| Isokinetic 120°s Total Work (J) | 21 | 500.60±272.72 | 23 | 369.87±160.76 |

Abbreviations: BW, body weight; COP, centre of pressure elliptical area; HGS, hand grip strength; PS, postural stability; PT, peak torque; STS-60, sit to stand 60; TUAG, timed up and go.

Table S3. Summary of physical activity variables values at baseline

| **Variable** | **n** | **Baseline (Mean ± SD) (Intervention)** | **n** | **Baseline (Mean ± SD) (Control)** |
| --- | --- | --- | --- | --- |
| ENMO (mg) | 18 | 25.23±8.76 | 19 | 27.31±8.93 |
| Inactivity (min) | 15 | 660.70±129.21 | 18 | 650.94±101.54 |
| Light activity (min) | 17 | 244.97±89.04 | 19 | 231.84±51.27 |
| Moderate activity (min) | 17 | 82.47±52.83 | 19 | 95.52±49.83 |
| Vigorous activity (min) | 17 | 3.20±5.97 | 19 | 2.27±2.49 |

Abbreviations: ENMO, Euclidean norm minus one

Table S4. Summary of body composition variables values at baseline

| **Variable** | **n** | **Baseline (Mean ± SD) (Intervention)** | **n** | **Baseline (Mean ± SD) (Control)** |
| --- | --- | --- | --- | --- |
| Weight (kg) | 22 | 81.55±21.59 | 24 | 80.55±13.07 |
| Body fat (%) | 22 | 35.71±9.29 | 24 | 34.54±9.75 |
| Skeletal MM (kg) | 22 | 28.30±7.69 | 24 | 28.85±5.56 |
| Fat-free mass (kg) | 22 | 51.84±13.06 | 24 | 52.17±9.39 |
| Lean mass (kg) | 22 | 48.73±12.38 | 24 | 49.15±8.90 |
| Fat mass (kg) | 22 | 29.71±12.79 | 24 | 28.38±10.15 |

Abbreviations: MM, muscle mass

Table S5. Summary of metabolic and inflammatory markers at baseline

| **Variable** | **n** | **Baseline (Mean ± SD) (Intervention)** | **n** | **Baseline (Mean ± SD) (Control)** |
| --- | --- | --- | --- | --- |
| Glucose (mmol/L) | 22 | 5.83±2.93 | 23 | 6.44±1.44 |
| HbA1c (%) | 22 | 6.04±1.40 | 22 | 6.12±0.86 |
| HbA1c (mmol/mol) | 22 | 42.68±15.23 | 22 | 43.41±9.42 |
| Cholesterol (mmol/L) | 22 | 4.34±0.88 | 24 | 4.47±1.09 |
| Triglycerides (mmol/L) | 22 | 1.60±0.80 | 24 | 2.09±1.28 |
| HDL (mmol/L) | 21 | 1.39±0.29 | 24 | 1.37±0.41 |
| LDL (mmol/L) | 21 | 2.30±0.77 | 23 | 2.13±0.85 |
| IL-6 (pg/mL) | 9 | 1.42±0.91 | 11 | 1.51±1.30 |
| IL-10 (pg/mL) | 4 | 0.22±0.22 | 6 | 0.19±0.07 |
| TNF-α (pg/mL) | 4 | 1.63±3.06 | 6 | 1.68±3.58 |
| CRP (mg/L) | 12 | 3.33±2.69 | 16 | 2.56±2.39 |

Abbreviations: CRP, C-reactive protein; HDL, high-density lipoprotein; HbA1c, glycated haemoglobin; IL, interleukin; LDL, low-density lipoprotein; TNF, Tumor necrosis factor

Table S6. Summary of MRI and ultrasound thigh measurements at baseline

| **Variable** | **n** | **Baseline (Mean ± SD) (Intervention)** | **n** | **Baseline (Mean ± SD) (Control)** |
| --- | --- | --- | --- | --- |
| Ultrasound |  |  |  |  |
| RF-CSA (cm²) | 22 | 5.82±1.48 | 24 | 6.97±2.30 |
| RF-CSA/Ht² | 22 | 2.05±0.46 | 24 | 2.46±0.74 |
| RF-CSA/BMI | 22 | 0.21±0.06 | 24 | 0.25±0.10 |
| RF thickness (mm) | 22 | 22.56±5.26 | 24 | 22.70±5.23 |
| RF fat thickness (mm) | 22 | 15.16±8.34 | 24 | 12.90±6.86 |
| VL thickness (mm) | 21 | 27.27±6.99 | 24 | 25.14±7.14 |
| VL fat thickness (mm) | 21 | 10.65±7.12 | 24 | 9.43±5.63 |
| MRI |  |  |  |  |
| Quadriceps total volume (cm²) | 21 | 1044.76±346.04 | 22 | 1086.43±312.13 |
| Quadriceps volume/Ht² | 21 | 361.38±93.21 | 22 | 376.19±86.51 |
| Quadriceps volume/BMI | 21 | 35.94±8.65 | 22 | 39.02±13.33 |
| Quadriceps CSA (cm²) | 21 | 54.18±15.80 | 22 | 56.46±11.64 |
| Quadriceps CSA/Ht² | 21 | 18.83±4.42 | 22 | 19.74±3.34 |
| Quadriceps CSA/BMI | 21 | 1.87±0.40 | 22 | 2.00±0.46 |

Abbreviations: CSA, cross-sectional area; RF, rectus femoris; VL, vastus lateralis

Table S7. Summary of cardiac MRI variables at baseline

| **Variable** | **n** | **Baseline (Mean ± SD) (Intervention)** | **n** | **Baseline (Mean ± SD) (Control)** |
| --- | --- | --- | --- | --- |
| Left end diastolic volume (LVED, ml) | 25 | 140±42.8 | 22 | 147±31.6 |
| Left ejection fraction (%) | 25 | 64.3±10.3 | 22 | 63.7±8.2 |
| Left mass index (g/m^2^) | 25 | 66.5±16.8 | 22 | 71.3±17.9 |
| Left mass/LVED (g/ml) | 25 | 0.95±0.38 | 22 | 0.89±0.15 |
| Myocardial global native T1 time (ms) | 25 | 1280±35.9 | 22 | 1260±57.3 |
| Myocardial perfusion reserve (ml/min/g) | 25 | 2.2±0.6 | 22 | 3.2±0.7 |
| Right ejection fraction (%) | 25 | 55.8±9.9 | 22 | 55.6±7.6 |

Abbreviations: LVED, left ventricular end diastolic;

Table S8. Summary of self-reported measurements at baseline

| **Variable** | **n** | **Baseline (Intervention)** | **n** | **(Control)** |
| --- | --- | --- | --- | --- |
| PSQI global score | 24 | 8.58±5.33 | 23 | 7.65±5.04 |
| PAM score | 24 | 66.47±15.47 | 25 | 66.12±14.64 |
| DASI METs | 23 | 8.03±1.83 | 25 | 7.76±1.79 |
| Fatigue score (FACIT-F) | 24 | 112.02±33.55 | 25 | 115.79±28.59 |
| Health literacy  Low (n)  High (n) | 24 | 1  23 | 25 | 0  25 |
| SF-12 score MCS PCS | 24  24 | 46.12±13.82  46.90±11.56 | 25  25 | 47.77±12.15  45.86±10.16 |
| Symptoms experienced:  Pain (n)  Shortness of breath (n)  Weakness (n)  Nausea (n)  Vomiting (n)  Poor appetite (n)  Constipation (n)  Sore/dry mouth (n)  Drowsiness (n)  Poor mobility (n)  Itching (n)  Difficulty sleeping (n)  Restless legs (n)  Changes in skin (n)  Diarrhoea (n)  Transplant specific:  Headache (n)  Increased appetite (n)  Weight gain (n)  Weight loss (n)  Bloating (n)  Tremor (n)  Muscle soreness, aches, pains (n)  Pain bone and joints (n)  Poor libido (n)  Guilt (n)  Dissatisfaction with body image (n) | 21 | 11  12  15  4  2  9  6  7  8  10  7  16  8  5  3  11  13  16  6  16  17  13  16  13  12  18 | 22 | 16  16  20  3  2  2  7  7  10  8  9  15  7  6  5  13  13  17  3  9  14  14  14  6  8  19 |

Abbreviations: DASI, duke activity status index; MCS, mental component summary; METs, metabolic equivalents; PAM, patient activation measure; PCS, physical component summary; PSQI, Pittsburgh sleep quality index;


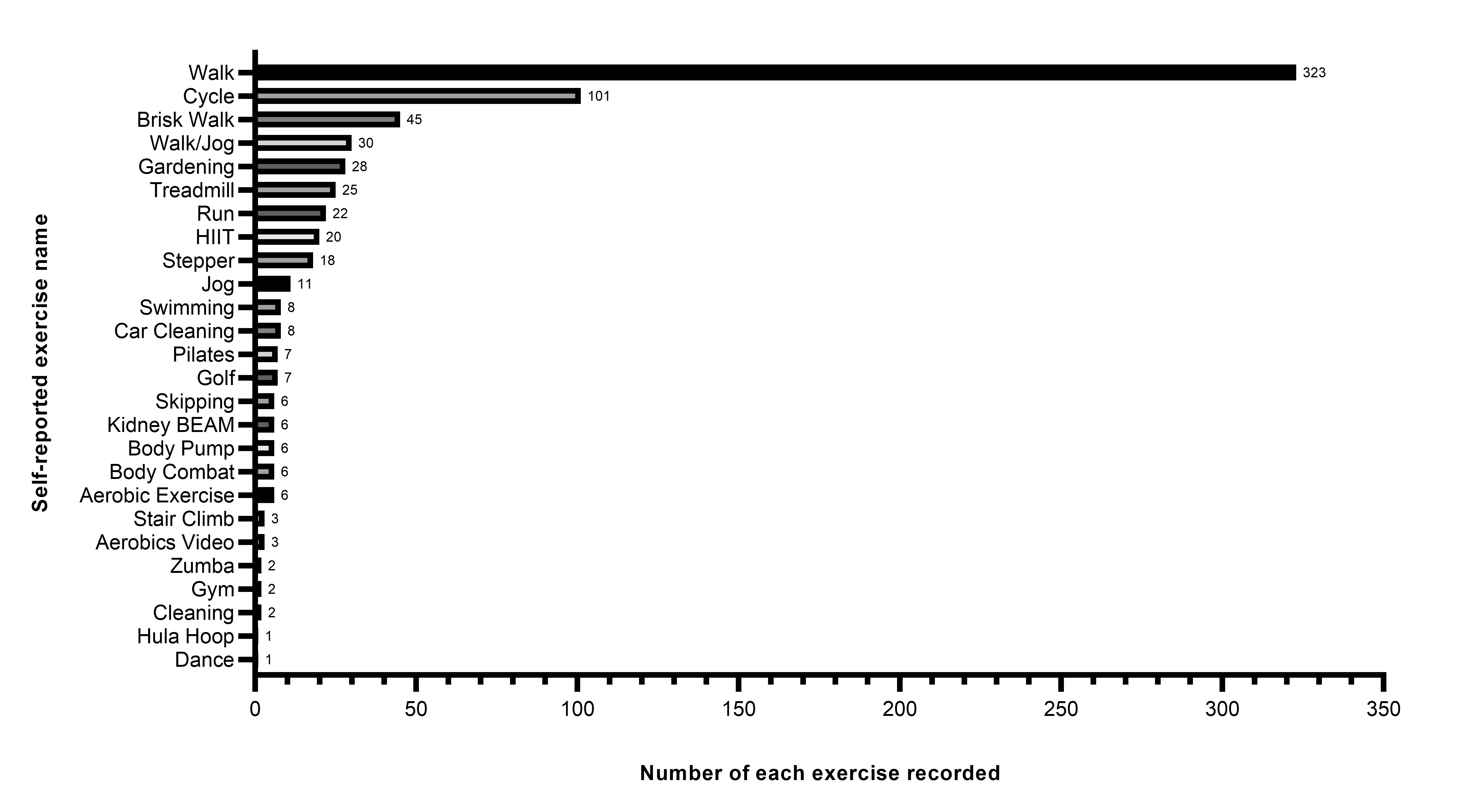


Figure S1. Self-reported aerobic exercise types and the total number of each reported over the 12-week intervention period.


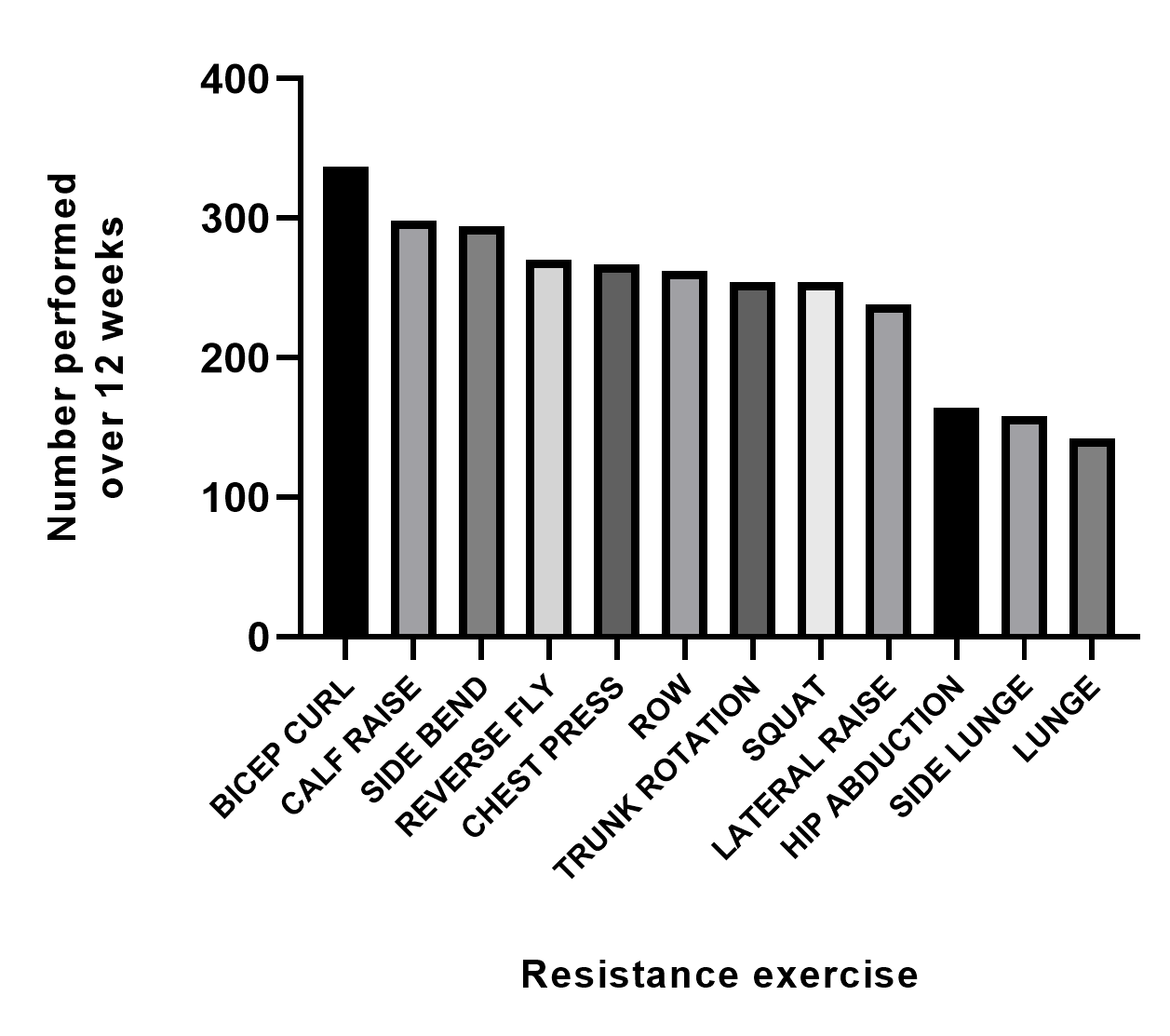


Figure S2. Total number of each resistance exercise performed over the 12-week programme.

Table S9. Free-text responses detailing the hardest and the most enjoyable aspects of the programme to participants.

| **Most enjoyable** | **Hardest** |
| --- | --- |
| Seeing strength develop | New routine |
| Seeing improvement | Personal motivation x2 |
| Everything – motivational phone calls | Reverse fly exercise x2 |
| Materials supplied to support the exercise | Home-based, struggled with commitment |
| Different physical activities | Weights x3 |
| Weights x6 | Too tired to do weights |
| Sweating and losing weight | Knee and shoulder problems with weights |
| Aerobic exercises | Pain from other issues |
| Walking and running | Finding the time |
| Feeling energised and healthier and seeing progress on my body | Finding the time, especially around appointments |
| I was able to do it on my own | Cardio – stamina |
| Getting stronger arms and chest | Injury |
